# Dreimuskelchirurgie bei großwinkliger Esotropie

**DOI:** 10.1007/s00347-020-01318-9

**Published:** 2021-01-20

**Authors:** Michael Gräf, Julia Röhm, Heiko Wassill

**Affiliations:** 1 Fachbereich Humanmedizin, Justus-Liebig-Universität Gießen, Gießen, Deutschland; 2 Klinik und Poliklinik für Augenheilkunde, Universitätsklinikum Gießen und Marburg, Standort Gießen, Friedrichstr. 18, 35392 Gießen, Deutschland

**Keywords:** Dosierung, Gesundheitsökonomie, Konvergenzoperation, Strabismus, Therapie, Grading, Health economy, Operation, Strabismus, Therapy

## Abstract

**Hintergrund:**

Zur Korrektur großwinkliger Esotropie sind die kombinierte Konvergenzoperation und die beidseitige Medialisrücklagerung ohne und mit Myopexie verbreitet. Nur wenige Berichte liegen zu Dreimuskeleingriffen (3 ME) vor. Wir analysierten die Ergebnisse von 3 ME.

**Patienten und Methoden:**

Von Juni 2016 bis Mai 2020 erhielten 61 Patienten einen 3 ME wegen Esotropie ≥ 27°. Schrägschielen wurde mitbehandelt. Die Schielwinkel wurden in 5 m und 0,3 m im simultanen (SPCT) und alternierenden Prismenabdecktest (APCT) gemessen. Die Dosierung betrug 0,51 mm/° (APCT, 5 m). Die Ergebnisse von 57 Patienten waren auswertbar.

**Ergebnisse:**

Die Mediane und Streubreiten (min-max) betrugen: Alter: 6 Jahre (3–56). APCT präoperativ: fern 34° (27–45), nah 36° (27–50). Operationsdosis: 17 mm (15–21), 21 Fälle mit Obliquuschirurgie. APCT 5 Monate (3–24) postoperativ: fern 2° (−10–18), nah 2° (−8–18). Einen Restwinkel im Betrag ≤ 6° (≈ 10 PD) wiesen (APCT) fern 39 (68 %), nah 38 (67 %) und (SPCT) fern 45 (79 %), nah 42 Patienten (74 %) auf. Eine konsekutive Exotropie > 6° im APCT hatten fern 4 (7 %), nah 3 (5 %), eine Esotropie > 6° hatten 14 (25 %) bzw. 16 Patienten (28 %).

**Schlussfolgerung:**

Die 3 ME ist als Ersteingriff bei großwinkliger Esotropie gut geeignet.

## Abstract (en)

**Background:**

Bilateral medial rectus muscle recession with or without Cüppers’ posterior fixation suture and recess and resect surgery are used to correct for large angle esotropia. There are only few reports on three muscle surgery (3MS). We analyzed the results of 3MS.

**Patients and methods:**

Between June 2016 and May 2020, 61 patients received 3MS for esotropia ≥ 27° (50 PD) together with oblique muscle surgery, if needed. Angles of strabismus were measured by simultaneous prism and cover testing (SPCT) and alternating prism and cover testing (APCT) at 5 m and 0.3 m. Grading was around 0.51 mm/degree (at 5 m). Medium-term results of 57 patients were available.

**Results:**

Medians and ranges (min-max) were: age, 6 years (3–56 years). Preoperative APCT, far 34° (27–45°), near 36° (27–50°). Amount of surgery, 17 mm (15–21 mm), oblique muscle recession in 21 cases. The APCT after 5 months (3–24 months), far 2° (−10–18), near 2° (−8–18). Success rates (absolute deviation ≤ 6° [10 PD]), APCT far 68%, near 67%, SPCT far 79%, near 74%. Exotropia > 6° occurred in 4 cases (7%) at far and 3 (5%) at near, esotropia > 6° in 14 cases (25%) at far and 16 (28%) at near.

**Conclusion:**

The use of 3MS is a suitable first step procedure to correct for large angle esotropia.

Esotropie mit und ohne Symptome eines frühkindlichen Schielsyndroms ist in Europa die häufigste primäre Schielform. Ihre Behandlung hängt von der Größe des Schielwinkels, dem Vorhandensein von Konvergenzexzess, Kopfzwangshaltung, dissoziierten Komponenten und Obliquussstörungen, einer Amblyopie, dem Patientenalter und der lokal etablierten Vorgehensweise ab. Zur Korrektur großer Schielwinkel ist im deutschsprachigen Raum eine Zweischrittstrategie mit einer kombinierten Konvergenzoperation (KK) als Ersteingriff verbreitet, im angelsächsisch beeinflussten Raum mehr die beidseitige Medialisrücklagerung (BMR) [14, 24]. Im Fall eines starken Effekts kann sich der zweite Eingriff erübrigen. Wünschenswert ist jedoch die Korrektur möglichst vieler Fälle in nur einer Operation. Hierzu stehen Drei- und Viermuskeleingriffe (3 ME, 4 ME) [4–6, 8, 20, 22, 26, 29] und die entweder hoch dosierte [19, 23, 30, 33–35] oder durch eine beidseitige Fadenoperation (BMRF) verstärkte BMR zur Verfügung [11, 15, 16]. Sehr hoch dosierte KK verursachen ein Adduktionsdefizit mit horizontaler Inkomitanz, die nur unter bestimmten Bedingungen wie funktioneller Einäugigkeit akzeptabel ist [17, 31]. Hoch dosierte BMR von 7–8 mm hinterließen in vielen Fällen eine residuelle Esotropie [1, 7, 23, 30]. Im Säuglingsalter war allerdings bei Kontrollen nach 2 Jahren eine Exotropie häufig die Folge [28]. Die BMRF ist technisch anspruchsvoller und schwieriger revidierbar. Zur Alternative der Y‑Split-BMR bei großer Esotropie existieren nur wenige Berichte [2, 3, 13].

Die Dreimuskelchirurgie in Form der BMR mit einseitiger Lateralisverkürzung ist eine interessante Alternative. Die Rücklagerungen überschreiten dabei selten den Bulbusäquator. Die Technik ist unkompliziert. Auch eine Verstärkung oder die Abschwächung eines Übereffekts ist relativ einfach. Die Studienlage zu 3 ME ist jedoch nicht einheitlich [4–6, 8, 20, 22, 26, 29]. Die unseres Wissens einzige systematische Evaluation im deutschen Sprachraum umfasst 27 Patienten, die von 2003 bis 2008 an 4 Orten in der Schweiz operiert wurden [29]. Der Schielwinkel wurde im Mittel von ca. 30° auf 5,5° verkleinert. Einen Restschielwinkel im Betrag (Absolutwert) von ≤ 10 PD (5,8°) wiesen nach 6 Monaten 64 % der Patienten auf. In anderen Arbeiten sind Erfolgsraten von 30 % [22] bis 92 % [26] genannt, in der Studie mit dem größten Gruppenumfang 62 % nach teilweise langem Follow-up [6]. Wir wenden 3 ME seit Juni 2016 zur Behandlung großwinkliger Esotropie an. Diese Studie dient zur Kontrolle der Ergebnisqualität.

## Patienten und Methoden

### Patienten

Mit Zustimmung der lokalen Ethikkommission wurden alle 61 Patienten erfasst, die von Juni 2016 bis Mai 2020 wegen einer konkomitanten Esotropie mit oder ohne vertikale Inkomitanz nach ausführlicher Aufklärung über das operative Vorgehen und schriftlicher Einverständniserklärung einen 3 ME mit oder ohne Obliquuschirurgie erhielten. Einschlusskriterium war ein Schielwinkel ≥ 27° (50 PD) im Fernblick. Zwei Patienten waren wegen Esotropie an den Mm. recti mediales voroperiert. Sie wurden im Interesse einer kompletten Darstellung in die Auswertung genommen, aus der Berechnung der Effektivität der 3 ME (°/mm-Koeffizienten) jedoch ausgeschlossen. Generelle Ausschlusskriterien waren motorischer Nystagmus, paretisches Schielen, hohe Myopie und endokrine Orbitopathie. Ein Patient wurde deshalb nicht in die Auswertung genommen. Drei Patienten kamen nicht zur Kontrolle (Rücklaufquote 95,0 %). Die Daten von 57 Patienten wurden evaluiert. Die Maßgaben der Deklaration von Helsinki wurden beachtet.

### Diagnostik

Die Schielwinkel wurden im simultanen und alternierenden Prismenabdecktest (SPCT, APCT), jeweils mit Brillenkorrektion (Refraktion in Zykloplegie abzüglich 0,5 dpt vom sphärischen Wert) bei Fixation eines Lichts in 5 m und eines kleinen, Akkommodation fordernden Objekts in 0,3 m Abstand gemessen. Zur Messung großer Schielwinkel befanden sich ein 27°-Prisma vor dem abgewichenen Auge und ein Prisma zur völligen Neutralisierung des Winkels vor dem fixierenden Auge. Da die Messprismen in Grad kalibriert waren, entsprach die Summe beider Werte nach Addition der prismatischen Effekte der Brillengläser dem Schielwinkel [9, 12]. Postoperativ, vor Entlassung und bei der letzten Visite am Ende der Nachbeobachtungszeit von individuell 3 bis 10 (in 6 Fällen 12 bis 24) Monaten, genügten Einzelprismen. Die axiale Bulbuslänge wurde mit dem IOL-Master 500 (Carl Zeiss, Jena) bestimmt. Das postoperativ vorhandene Binokularsehen wurde klassifiziert in Suppression (0), Simultansehen im Bagolini-Lichtschweiftest (1), Titmus-Fliege (2), Titmus/Randot-Ringe oder -Tiere (3) und Random-Dot-Stereosehen im Lang-1-Test (4). Die Diagnose der Schielform basierte auf dem anamnestischen Schielbeginn, der Refraktion und dem nachgewiesenen Binokularsehen.

### Operatives Vorgehen

Alle Eingriffe erfolgten in Vollnarkose unter stationären Bedingungen. Die Dosierung D war unter Beachtung der axialen Bulbuslänge BL auf den Fernschielwinkel F bezogen (Richtwert: D = 0,5 mm/° × BL/22/mm × F/°). Die Eingriffe erfolgten über Limbusschnitte mit radiären Erweiterungen oder nur über radiäre Schnitte, die Muskelnähte mit ¼‑Kreis-Spatula-armiertem 6–0-Polyglactin, der Wundverschluss mit 9–0-Polyglactin. Zur Rücklagerung wurde der sklerale Einstichpunkt mit einem Messzirkel um den Betrag der Rücklagerung weiter vom Limbus entfernt markiert als die das obere und untere Sehnenviertel greifenden Schlingen vor Desinsertion der Sehne lagen. Zur Faltung wurden das obere und untere Muskeldrittel angeschlungen, die Fäden dicht an der Insertion durch die Sklera geführt und die Falte mit einem Irisspatel zwischen Muskel und Sklera flach ausgespannt [18]. Eingriffe an den Mm. obliqui erfolgten mit dem gleichen Material über denselben, am Auge mit der alleinigen Medialisrücklagerung über einen distalen radiären Schnitt.

### Auswertung

Erfasst wurden das Alter bei Operation, der Operateur, die Operationsstrecke pro Muskel und die horizontalen Fern- und Nahschielwinkel präoperativ (APCT), vor Entlassung und bei der letzten Visite ≥ 3 Monate postoperativ (SPCT, APCT). Zudem wurde die postoperative Binokularfunktion bei der letzten Visite analysiert.

### Statistik

Streubreiten und Quartile, für den Literaturvergleich auch Mittelwerte und Standardabweichungen sowie der °/mm-Koeffizient als Maß für die Effektivität der 3 ME, bezogen auf die Winkel im APCT, wurden ermittelt – dies ohne die Daten der 2 voroperierten Patienten, bei denen das Drehmoment der weit dorsal verankerten Recti mediales zusätzlich durch den kürzeren Hebelarm reduziert war. Als statistischer Test für Gruppenvergleiche diente der Mann-Whitney-U-Test.

## Ergebnisse

### Allgemeine Daten

Die 61 Eingriffe in den Jahren 2016 (2), 2017 (7), 2018 (25), 2019 (23) und 2020 (4; COVID-Restriktionen) erfolgten durch 2 Operateure (MG, HW). Das Alter (im Folgenden jeweils Median und Streubreite) der 57 ausgewerteten Patienten (27♂, 30♀) betrug 6 Jahre (3–56). Die Tab. 1 zeigt die individuellen Patientendaten.

**Table 1:** 

Nr	Alter Jahre	Diagnose	BL mm	SE dpt	APCT F prä Grad	APCT N prä Grad	Operationsdosis Muskeln, mm	APCT F post Grad	APCT N post Grad	SPCT F post Grad	SPCT N post Grad	BS Klassen	NBZ Monate
1	31	DEP	23,8	−1,0	30	28	KK57MR5	4	4,5	0	0	4	4
2^a^	24	FET	22,4	0	42	40	KK79MR6bOIR	16	17	16	17	0	6
3	3	FET	22,5	+1,5	38	40	KK57MR6bOIR8	2,5	9	2,5	9	0	14
4	6	AET	20,6	+6,75	33	33	KK57MR5	2	5	2	2	1	4
5	9	FET	–	−0,5	45	50	KK57MR6	8	8	8	8	0	6
6	4	FET	21,4	1,0	41	41	KK68MR6	4	0	3	0	0	5
7	6	FET	21,4	1,75	36	36	KK58MR5bOIR10	9	3	9	3	0	5
8	5	FET	23,2	+1,5	40	44	KK68MR6	−2	0	–	–	0	5
9	13	DEP	24,3	+0,5	32	34	KK57MR5	1	1,5	0	0	4	6
10	29	FET	22,9	+1,75	37	37	KK67MR6	4	1	4	0	1	3
11	5	FET	21,9	−2,0	29	30	KK57MR5OIR8	−1	−4	0	−4	0	4
12	5	FET	20,9	+2,75	35	35	KK57MR5	6	12	3	5	1	5
13	4	FET	22,0	+1,5	38	45	KK67MR6	1	5	1	5	0	24
14	6	AET	22,6	+5,25	35	38	KK65MR6	1	0	−2	0	1	3
15	5	FET	22,2	+0,75	30	32	KK57MR5	5	4	5	4	0	7
16	5	FET	21,8	+4,25	31	38	KK56MR5bOIR10	−2	2	−2	2	0	6
17	6	FET	22,1	+1,5	32	39	KK57MR5	0	0	0	0	3	3
18	9	FET	23,0	+1,5	35	36	KK56MR5	2	2	2	2	0	3
19	5	FET	21,9	+3,0	32	33	KK56MR5bOIR10	4	4	4	3	1	4
20	6	AET	19,5	+7,25	36	34	KK57MR5	8	1	8	1	1	14
21	6	FET	20,8	+4,0	34	46	KK57MR5	1,75	−2	3	4	1	4
22	9	FET	23,3	−0,75	32	37	KK56MR5	18	18	18	18	0	4
23^a^	5	FET	22,4	+2,25	34	35	KK57MR5	14	14	18	18	0	9
24	24	FET	21,8	+2,5	32	34	KK57MR6OIR10	1	1	1	1	1	7
25	4	FET	22,6	+0,25	35	37	KK66MR6	8	5	9	9	1	3
26	4	AET	20,0	+8,5	41	43	KK66MR6bOIR10	2	7	5	7	1	4
27	11	NSS	–	+1,25	34	44	KK67MR6	0	0	0	0	4	4
28	10	AET	20,4	+5,25	35	32	KK56MR5bOSR8	9	7	0	3	0	7
29	4	NSS	20,4	+3,25	30	35	KK56MR5bOIR10	2	6	0	1	3	7
30	5	FET	22,7	0	35	35	KK57MR6bOIR10	5	10	5	10	1	14
31	3	DMS	21,6	+0,75	33	35	KK57MR5	−5	−2	−3	−3	–	6
32	5	FET	21,4	+2,25	30	27	KK55MR5	−8	−2	−5	−3	1	15
33	3	FET	21,3	+2,0	41	35	KK67MR6	14	12	14	12	–	3
34	3	FET	21,5	+1,25	30	27	KK56MR5	11	12	1	3	0	3
35	21	DMS	22,0	+0,25	36	41	KK57MR6	0,5	0,5	0,5	1	1	4
36	13	DEP	23,0	+0,75	36	43	KK66MR6	0	0	2	0	4	3
37	7	NSS	22,5	+3,0	32	35	KK57MR5OIR6	0,5	0,5	2	2	3	10
38	3	FET	20,8	+2,5	35	39	KK57MR5	11	16	10	16	0	4
39	10	FET	21,9	+1,25	33	33	KK55MR5	8	7	4	3	0	6
40	4	AET	20,6	+7,0	36	41	KK56MR5OIR8	−1,5	0,5	−3	−3	0	5
41	4	FET	21,8	+3,25	36	36	KK57MR5bOIR10	1	1	3	3	1	3
42	23	DEP	23,8	−1,0	29	35	KK56MR5	7	4,5	0	0	4	8
43	7	FET	22,0	0,25	32	41	KK56MR5	−3	15	0	15	2	12
44	6	FET	22,6	+0,25	32	32	KK57MR5	−2	−4	−2	−4	0	3
45	6	FET	22,4	+0,25	35	35	KK57MR6	0	−5	0	−2	0	3
46	4	AET	20,5	+4,25	30	37	KK56MR5bOIR10	9	10	5	10	0	4
47	3	FET	22,8	+0,75	38	45	KK76MR6bOIR10	−6	−6	−6	−6	0	3
48	3	AET	20,1	+6,75	30	38	KK57MR5bOIR10	−8,5	−6,5	−7	−5	0	6
49	6	SET	20,3	+10,0	30	30	KK54MR6bOIR10	−5	−8	−5	−8	0	6
50	5	FET	22,2	+2,75	27	33	KK55MR5	1	8	1	8	0	7
51	38	SET	22,7	−0,25	27	27	KK56MR5	−2	−3	0	0	3	4
52	6	FET	20,9	+5,0	29	36	KK56MR5bOIR8	2	2	0	0	0	3
53	5	AET	20,3	+5,5	34	38	KK65MR6bOIR10	0,5	−3	0,5	−3	4	4
54	8	FET	20,8	+3,0	38	39	KK58MR6	−10	−7,5	−10	−7,5	0	6
55	6	FET	23,9	+2,5	34	46	KK65MR6	−10	1	−10	1	1	3
56	56	AET	22,0	+5,0	44	44	KK78MR5 rev	1,5	1,5	2	2	1	5
57	38	DMS	26,5	−4,0	36	44	KK37MR6 rev	2	3	0	0	3	3

*DEP* dekompensierte Esophorie, *FET* frühkindliche Esotropie, *AET* fraglich akkommodativ entstandene Esotropie, *DMS* dekompensierter Mikrostrabismus, *SET* sekundäre Esotropie, *NSS* normosensorisches Spätschielen, *BL* axiale Bulbuslänge (Mittelwert beider Augen), *SE* sphärisches Äquivalent (Mittelwert beider Augen), *Ferne/Nähe* Fern/Nahschielwinkel, *prä/post*: prä-/postoperativ, *KK* kombinierte Konvergenzoperation, *MR* kontralaterale Medialisrücklagerung, *OIR/OSR* Obliquus-inferior/superior-Rücklagerung, *b* beidseitig, *BS* Binokularsehen, *NBZ* Nachbeobachtungszeit = Zeitpunkt der letzten Visite, *rev* voroperierter Patient, *F* Ferne, *N* Nähe

^a^Patient mit späterem zweitem Eingriff

### Operation

Die Operationsstrecke an den Horizontalmotoren betrug 17 mm (15–21), bei einer relativ konstanten Dosierung von 0,51 mm/° ± 0,04 mm/°, somit ca. 0,5 mm Gesamtstrecke pro Grad Innenschielwinkel, annähernd gleich verteilt auf die 3 Muskeln. Wegen eines Strabismus sursoadductorius erhielten 15 Patienten zusätzlich beidseitig, 5 einseitig eine Rücklagerung des M. obliquus inferior. Bei 1 Patient erfolgte wegen eines Strabismus deorsoadductorius beidseits eine Rücklagerung des M. obliquus superior. Bei den 2 voroperierten Patienten erfolgten Rücklagerungen von 9 auf 14 mm und 7,5 auf 12,5 mm Limbusabstand (Tab. 1, Nr. 57) bzw. von 10,5 auf 13,5 mm am schon operierten Auge (Nr. 56).

### Schielwinkel

**Figure 1:**
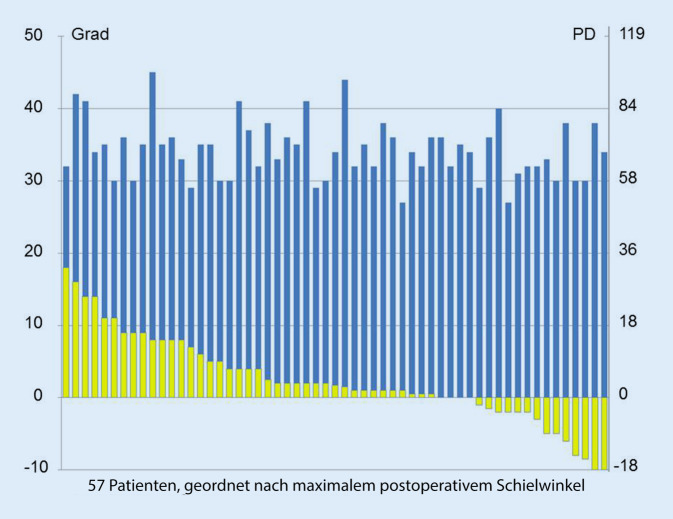

Die Schielwinkel von präoperativ (APCT) fern 34° (27–45), nah 36° (27–50) waren am Ende der Nachbeobachtungszeit von 5 (3–24) Monaten auf (APCT) 2° (−10–18) bzw. 2° (−8–18) reduziert und im SPCT auf 1° (−10–18) bzw. 2° (−8–18). Direkt postoperativ war die Streuung etwas größer (Tab. 2). Klinisch sind v. a. die Ergebnisse nach ≥ 3 Monaten interessant. Die folgenden Angaben beziehen sich daher auf die Ergebnisse am Ende der Nachbeobachtungszeit, die in den meisten Fällen 3 bis 10 Monate betrug (*n* = 51) und in 6 Fällen 12 bis 24 Monate. Auf einen (absoluten) Winkelbetrag ≤ 5° im APCT waren fern 37 (65 %) und nah 36 (64 %) Patienten korrigiert, auf einen Betrag ≤ 6° (≈ 10 PD) 39 (68 %) bzw. 38 (67 %) Patienten. Im SPCT (*n* = 56) betrugen die entsprechenden Raten fern 43 (77 %), nah 40 (71 %) bzw. fern 44 (79 %) und nah 41 (73 %). Die Abb. 1 zeigt die individuelle Schielwinkelreduktion im APCT. Eine konsekutive Exotropie > 6° im APCT hatten fern 4 (7 %), nah 3 (5 %), eine Esotropie > 6° im APCT hatten 14 (25 %) bzw. 16 (28 %) Patienten, 2 erhielten einen verstärkenden Eingriff. Dieser korrigierte auf einen Restbetrag ≤ 3°.

**Table 2:** 

	MW	SD	Median	Minimum	Maximum
Alter (Jahre)	9,9	10,8	6	3	56
Sphärisches Äquivalent (D)	2,3	2,7	1,75	−4,0	10,0^a^
Axiale Bulbuslänge (mm)	21,9	1,3	21,9	19,5	26,5
Nachkontrolle (Monate)	5,8	3,9	5	3	24
Ferne präoperativ APCT (°)	34,3	4,1	34	27	45
Nähe präoperativ APCT (°)	37,0	5,2	36	27	50
Ferne 1 bis 2 Tage postoperativ APCT (°)	0,1	6,8	0	−18	16
Nähe 1 bis 2 Tage postoperativ APCT (°)	1,0	6,6	0	−11	19
Ferne final APCT (°)	2,6	6,1	1,875	−10	18
Nähe final APCT (°)	3,1	6,1	1,75	−8	18
Operationsstrecke (mm)	17,2	1,3	17	15	21
Operationseffekt (°/mm) *n* = 55	1,83	0,36	1,82	0,88	2,56
Ferne 1 bis 2 Tage postoperativ SPCT (°)	−0,1	6,3	0	−18	16
Nähe 1 bis 2 Tage postoperativ SPCT (°)	0,7	6,3	0	−11	19
Ferne final SPCT (°)	2,1	5,8	1	−10	18
Nähe final SPCT (°)	2,8	6,1	1,5	−8	18

*APCT* alternierender Prismenabdecktest, *SPCT* simultaner Prismenabdecktest, *MW* Mittelwert, *SD* Standardabweichung, *D* Dioptrie (MW beider Augen)

^a^Durch einseitige Aphakie (18,5 D)

### Konvergenzexzess

Eine Differenz von > 5° zwischen Nah- und Fernschielwinkel wiesen 14 Patienten auf. Postoperativ zeigten noch 3 dieser 14 sowie 2 weitere Patienten eine solche Differenz.

### Effektivität (°/mm-Koeffizient)

Die Schielwinkelreduktion bezogen auf die Gesamtdosis betrug direkt postoperativ durchschnittlich 2,0°/mm. Dieser Wert nahm bei den 27 Patienten mit Visite nach 3 bis 4 Monaten von 2,0° auf 1,8°/mm ab. Bei den 6 Patienten mit Visite nach 12 bis 24 Monaten nahm er von initial 1,9° auf 2,0°/mm zu. Bei den 20 Patienten mit Visite nach 5 bis 7 Monaten lag er konstant bei 1,9°/mm. Die mittlere Effektivität aller 55 primären 3 ME betrug am Ende der individuellen Nachbeobachtungszeit 1,8°/mm ± 0,4°/mm, wobei kein signifikanter Unterschied zwischen den Operateuren auffiel (*p* = 0,21).

### Binokularsehen

Bei der letzten Visite, die nach 3 bis 24, im Median nach 5 Monaten stattfand (Tab. 1), zeigten 26 (46 %) Patienten Suppression, 18 (32 %) Simultansehen im Bagolini-Test, 5 (9 %) mehr oder weniger grobes (Titmus-Test) und 5 feines (Lang-1-Test) Stereosehen. In 2 Fällen lag keine Dokumentation vor.

## Diskussion

In unserer Patientengruppe konnten großwinklige Esotropien in der Mehrzahl der Fälle durch 3 ME in einem einzigen Schritt korrigiert werden. Die Erfolgsraten bei der letzten Kontrolle betrugen 65 % und 69 % nach dem Kriterium eines maximalen Fernschielwinkels (APCT) innerhalb von ±5° bzw. zum Literaturvergleich ±6°. Sie unterschieden sich unwesentlich von der Rate von 67 % (innerhalb ±6°) am Entlassungstag. Im Gruppenmittel war bis zur jeweils letzten Visite eine Esodrift von ca. 2° erkennbar (Tab. 2). Auf einen manifesten Schielwinkel (SPCT) innerhalb von ±5° bzw. ±6° waren am Ende der individuellen Nachbeobachtungszeit 77 % bzw. 79 % der Patienten korrigiert. Die Werte sind vergleichbar mit unseren Korrekturraten nach BMRF bei großwinkliger Esotropie mit 69 % und den Resultaten der 3 ME mit 64 % in der Studie von Sturm et al. bei einer ähnlichen Nachbeobachtungsdauer [11, 29]. In ihrer Auswertung war der präoperative Schielwinkel in der Hälfte der Fälle < 27°, die Effektivität nach 6 Monaten betrug im Mittel 1,6°/mm. Ein deutlicher Übereffekt trat nur in einem von 27 Fällen auf [29]. Bei ähnlicher Dosierung betrug die Effektivität unserer 3 ME im vergleichbaren Zeitfenster um 6 Monate durchschnittlich 1,8°/mm. (Der unmittelbar postoperative Wert von 2,0°/mm entsprach zufällig der Dosierung von 0,5 mm/°.) In unserer doppelt so großen Gruppe zeigten 4 Patienten einen Übereffekt. Untereffekte waren häufiger (Abb. 1). Deshalb haben wir unsere Dosierung nach dieser Auswertung um 7 % erhöht.

Systematische Untersuchungen von 3 ME in Form von kleinen Fallserien bis hin zu prospektiven Studien datieren bis in die 1980er-Jahre. Beim Vergleich mit diesen Publikationen sind eine Reihe von Faktoren zu berücksichtigen, besonders das Alter der Patienten zum Zeitpunkt des Eingriffs und die damit einhergehenden anatomischen und physiologischen Unterschiede wie die geringere Muskellänge und Größe des Auges, die Konvergenzreserve und die Art der Schielwinkelmessung. Im Säuglingsalter ist die Messung fast nur, im Kleinkindalter oft nur anhand der Hornhautspiegelbilder möglich und dadurch ungenau und untersucherabhängig. Für wissenschaftliche Zwecke könnte eine fotografische Messung erfolgen, die auch aus der Ferne möglich ist. Der Messwert kommt dann annähernd dem Wert im SPCT gleich. Wenn die Messung, wie in dieser Altersklasse häufig, in der Nähe erfolgt, kann ein Konvergenzexzess den Winkel vergrößern. Es kann aber auch fehlende Naheinstellung zur Unterschätzung der Esotropie führen, gerade wenn ein Licht als Fixierobjekt dient. Das betrifft Messungen im Prismenabdecktest ebenso. Systematische Fehler sind möglich, wenn Schielwinkel von > 50 PD (27°) die Kombination von Prismen erfordern. Durch die Addition der PD-Werte zweier, vor beide Augen verteilter Prismen wird der tatsächliche Schielwinkel unterschätzt [25]. Um die vor der Addition erforderliche Umrechnung in Grad zu erübrigen, sind unsere Prismen in Grad kalibriert. Eklatante Fehler können durch die Addition der nominellen Werte aufeinander gehaltener Prismen oder durch falsche Haltung der Prismen entstehen [9, 18]. Außerdem ist die prismatische Ablenkung stärkerer Brillengläser zu addieren [9, 12, 32]. Den meisten Publikationen ist nicht zu entnehmen, ob diese Faktoren beachtet wurden. Intraoperativ kann die Messtechnik zu Abweichungen der tatsächlichen von der geplanten Dosis, besonders der Rücklagerungen, führen. Auch diesbezüglich fehlen in den meisten Publikationen exakte Beschreibungen. Weitergehende Kalkulationen von °/mm-Koeffizienten wären deshalb müßig. Postoperativ, bei kleineren Schielwinkeln und durch längere Nachbeobachtungszeit höherem Alter und daher besserer Kooperation von Kindern sind die Messwerte verlässlicher. Als brauchbarer Vergleichsparameter kann der Anteil gut korrigierter Patienten mit einem Betrag bzw. Absolutwert des Restschielwinkels von ≤ 6° (10 PD) dienen. Dabei sind schon die statistischen Vertrauensbereiche dieser Prozentraten beträchtlich. Für unsere Gruppe von 57 Patienten erstreckt sich das 95 %-Konfidenzintervall der Erfolgsrate von 68,4 % zwischen 54,6 % und 79,7 %. Für kleinere Kohorten resultieren noch größere Konfidenzintervalle. Angaben von Dezimalstellen erübrigen sich daher.

Lee und Dyer berichteten über 36 Kinder, die zum Teil sehr früh und zum Teil erst im Alter von ca. 2 Jahren 3 ME wegen infantiler Esotropie von > 27° (50 PD) erhielten [20]. Bei den 14 Kindern, die im Alter um 12 Monate operiert wurden, betrug der mittlere absolute Restschielwinkel 12°. Alle erhielten einen zweiten Eingriff, je zur Hälfte wegen Esotropie oder Exotropie, oft mit zusätzlicher Obliquuschirurgie, 6 benötigten eine dritte Operation. Die 22 Kinder, die im Alter um 23 Monate operiert wurden, wiesen einen absoluten Restschielwinkel von durchschnittlich 3,8° auf. Die Nachbeobachtungszeit betrug im Mittel 3 bzw. 4 Jahre. Die Autoren hielten einen Operationszeitpunkt für vorteilhaft, zu dem die für genaue Messungen erforderliche Kooperation gegeben ist [20].

Scott et al. fanden 31 von 48 Kindern (65 %), die wegen einer Esotropie von durchschnittlich 33° einen 3‑ oder 4 ME erhalten hatten (Altersmittel bei Operation: 2,7 Jahre), auf einen Absolutbetrag von ≤ 10 PD korrigiert, im Vergleich zu nur 37 % nach BMR [26]. Die durchschnittliche Nachbeobachtungszeit betrug 2,6 Jahre.

Forrest et al. berichteten über 49 Kinder, die im Alter um 13 Monate wegen infantiler Esotropie von > 60 PD (31°) operiert und im Fall eines Über- oder Untereffektes aggressiv mit Miotika oder Prismen nachbehandelt wurden. Zwei bis 6 Monate postoperativ waren 92 % auf einen Betrag von ≤ 10 PD korrigiert, nach 2 Jahren 80 %, nach 6 Jahren 71 %. Postoperative Eso- und Exotropie von > 10 PD waren gleich häufig [8]. In einer späteren Publikation mit demselben Seniorautor und 51 Patienten nannten Camuglia et al. phantastische Erfolgsraten von 100 % nach 2 Monaten und 96 % nach 6 Monaten [5]. Man kann annehmen, dass die Messgenauigkeit in der früh-postoperativen Phase allein schon altersbedingt begrenzt war, und die initialen Erfolgsraten als Melange aus Operationseffekt und Messbias interpretieren. Im Alter von 4 und 8 Jahren waren noch 78 % bzw. 74 % der Kinder korrigiert [5]. Zweiteingriffe erfolgten später in 18 %, gleich häufig wegen Eso- oder Exotropie.

Minkoff und Donahue sahen nach vergleichbar frühen Operationen bei einem Follow-up von 8 bis 70 Monaten (MW 3 Jahre) nur 3 von 10 Kindern korrigiert, 7 waren exotrop, 1 Kind war esotrop [22].

Chatzistefanou et al. werteten die Daten von 194 Patienten aus, die von 1973 bis 2008 wegen großwinkliger Esotropie (≥ 50 PD) einen 3 ME erhalten hatten [6]. Das Alter zum Zeitpunkt der Operation betrug 20 Monate bis 36 Jahre mit dem Median bei 2,7 Jahren. Der mittlere Schielwinkel im alternierenden Abdecktest am Synoptophor ist mit 68,2 PD (34°) angegeben, bei einer Streuung von 50 PD (27°) bis 100 PD (45°). Die Erfolgsraten betrugen 79 % nach 8 Wochen und 62 % zur finalen Kontrolle nach ≥ 6 Monaten (MW 7 Jahre) bei einem Patientenrücklauf von 100 % bzw. 92 %. Eine konsekutive Exotropie war nach 8 Wochen mit nur 5 % seltener, später mit 24 % häufiger als eine residuale Esotropie, deren Anteil von 15 % über die Zeit konstant blieb. In einer statistischen Analyse wurden großer präoperativer Schielwinkel und Vorliegen von Obliquusstörungen als Parameter mit einem negativen Einfluss auf das Operationsergebnis identifiziert [6].

Bayramlar et al. fanden bei teilweise älteren Kindern mit präoperativen Schielwinkeln von durchschnittlich 69 PD (35°) eine Erfolgsrate von 78 % nach 5 bis 63 Monaten. Deutlich unterkorrigiert waren 4 Patienten, ein wesentlicher Übereffekt wurde nicht beobachtet [4].

Im Vergleich zur BMRF, die bei dieser Indikation ähnliche Erfolgsraten lieferte [11], ist ein 3 ME weniger invasiv und technisch einfacher. Im Rahmen unserer Studie reduzierten 3 ME in vielen Fällen auch einen Konvergenzexzess. Allerdings bevorzugen wir bei ausgeprägtem Konvergenzexzess die BMRF. Bei den meisten der 14 Patienten mit einer Nah-Fern-Differenz des Schielwinkels von > 5° war der Konvergenzexzess relativ gering.

Zusammengefasst war mittel- bis langfristig ein zufriedenstellendes Ergebnis bei zwei Drittel bis drei Viertel der Patienten durch einen 3 ME zu verzeichnen. Bei der Bewertung dieser Studien, auch solchen zur BMR, ist das Operationsalter zu beachten. In vielen Ländern ist es üblich, den ersten Eingriff schon im Säuglingsalter, spätestens aber im 2. Lebensjahr durchzuführen. Ein wirklicher Vorteil dieses frühen Operierens ist aus den genannten Studien nicht erkennbar. Die Ergebnisse in der Altersklasse um 12 Monate scheinen stärker zu streuen als im Alter ab 2 Jahre bis ins Erwachsenenalter. Die als Argument für frühes Operieren angeführte Chance auf feines Stereosehen ist bei infantiler Esotropie gering, die erreichbaren Binokularfunktionen sind prinzipiell limitiert. Im deutschen Sprachraum ist daher ein eher abwartendes Verhalten verbreitet, so lange bis zuverlässige Visusangaben auch nach der Operation eine sichere Amblyopieüberwachung erlauben. Bis dahin wirkt ein großer Schielwinkel im Vergleich zum konsekutiven Mikrostrabismus gleichsam Amblyopie-protektiv [10]. Es ist dann auch leichter feststellbar, ob eine Obliquusstörung mit korrigiert werden muss. Simonsz und Kolling fanden in einer Metaanalyse der ELISSS (Early vs. Late Infantile Strabismus Surgery Study) und 12 weiterer Studien Reoperationsraten von 60–80 % nach Ersteingriffen im Alter um 1 Jahr gegenüber 25 % für Kinder, die erst im Alter um 4 Jahre wegen infantiler Esotropie operiert worden waren [27]. 3 ME bieten evtl. die Chance, diese Rate weiter zu senken. Bei vermutlich akkommodativ ausgelöster Esotropie nach Orthotropie im ersten Lebensjahr kann ein früher Eingriff sinnvoll sein [21].

Unsere Studie umfasst eine Altersklasse, in der schon prä-, besonders aber postoperativ eine genaue Schielwinkelmessung möglich ist. Aufgrund der hohen Rücklaufquote sind die Ergebnisse repräsentativ. Die Nachbeobachtungszeiten sind noch relativ kurz. Der Vergleich mit Zweimuskeleingriffen bei ähnlicher Ausgangslage und eine Analyse von Langzeitergebnissen sollten dieser Studie folgen.

## Fazit

Durch Dreimuskeleingriffe in Form einer beidseitigen Medialisrücklagerung und einseitigen Lateralisfaltung wurde konkomitantes Innenschielen in der Größenordnung von 27–40° in mehr als zwei Drittel der Fälle auf einen Betrag von maximal 6° (10 PD) reduziert. Ein Dreimuskeleingriff ist technisch einfach und hinterlässt günstige Bedingungen für evtl. Folgeoperationen. Aufgrund der relativ hohen Erfolgsrate bietet sich ein Dreimuskeleingriff als primäres Verfahren zur Korrektur großwinkliger Esotropie an.
